# Reference equations for spirometric indices from a sample of the general adult population in Nigeria

**DOI:** 10.1186/s12890-017-0390-x

**Published:** 2017-03-06

**Authors:** Ademola Emmanuel Fawibe, Louis O. Odeigah, Mohammed J. Saka

**Affiliations:** 1 Department of Medicine, University of Ilorin/University of Ilorin Teaching Hospital, Ilorin, Nigeria; 2 Department of Family Medicine, University of Ilorin Teaching Hospital, Ilorin, Nigeria; 3 Department of Epidemiology and Community Medicine, University of Ilorin/University of Ilorin Teaching Hospital, Ilorin, Nigeria; 4 Department of Medicine, Faculty of Clinical Sciences, College of health Sciences, University of Ilorin, Ilorin, Kwara State Nigeria

**Keywords:** Reference equations, Spirometry indices, Pulmonary function tests, Nigeria

## Abstract

**Background:**

The increasing importance of pulmonary function testing in diagnosing and managing lung diseases and assessing improvement has necessitated the need for locally derived reference equations from a sample of the general Nigerian population.

**Methods:**

It was a cross sectional study in which we used linear regression models to obtain equations for reference values and lower limits of normal for spirometric indices in adult Nigerians from a sample of the general population aged 18–65 years (males) and 18–63 years (females).

**Results:**

Seven hundred and twenty participants made up of 358 males and 362 females who satisfactorily completed the spirometric measurements using the ATS/ERS reproducibility and acceptability criteria were included in the analysis. The most important predictive variables were height and age. The values of the spirometic indices increase with increasing stature but decrease with increasing age in both sexes. The sex difference in all the indices is also apparent as all the indices, except FEV_1_/FVC, are higher in men than in women.

Our values are higher than values obtained from previous studies in Nigeria (except FEV_1_/FVC) but the differences were not statistically significant. This suggests that although the values are increasing, the increase is yet to be significantly different from values obtained using the past equations. The implication of this is that there is need for periodic study to derive new equations so as to recognise when there is significant difference.

There was no significant difference between values from our equations and those obtained from study among Ethiopians. Compared to report from Iran, our FVC and FEV_1_values (in males and females) as well as PEFR (in females) are significantly lower. Our values are also lower than values from Poland. We also observed disparities between our values and those of Afro Americans from the GLI study.

**Conclusions:**

Our findings show that it is important to always interpret ventilatory function tests in any individual by comparing it with reference values obtained from a well-defined population of healthy subjects of the same ethnic origin in similar geographic location.

## Background

Over the time, pulmonary function tests have evolved from tools for physiologic study to clinical tools widely used in assessing the respiratory status. Spirometry is an important pulmonary function test for assessing ventilatory function of the lungs. The measurement of ventilatory indices is very important in the identification and management of respiratory diseases. Pulmonary function tests have also become a part of routine health examination in respiratory, occupational, and sports medicine. However, the results of pulmonary function tests should be interpreted in relation to reference values [1, 2].

Reference data are important for interpreting pulmonary function test results and can aid in the management of respiratory diseases. Factors such as age, sex, height, and race or ethnic origin have been shown to be important determinants of pulmonary function measurements [1–4]. Therefore, reference equations incorporating these factors have been used in North America [5, 6] and Europe [7–9] for several decades with some as far back as the sixties and seventies. These reference equations derived from surveys conducted in these regions are of limited use in Nigerians because of ethnic and regional differences in pulmonary function [10]. Even the recently published multi-ethnic global lung function 2012 equations are not useful in Nigerians because it did not include participants from Nigeria [11].

Ideally, reference values should be derived from a healthy and representative population tested by standardized technical methods and subjected to appropriate statistical analysis. Many of the older studies on pulmonary function in Nigerians fail to meet these criteria [12–16]. Almost all the studies are not representative of the general population. In some, the studies were restricted to students and staff of schools and hospitals [12, 13, 16]. None of the studies was done in more than one city/location and none involved participants from rural communities. The increasing importance of pulmonary function testing in diagnosing and managing lung diseases and assessing improvement has necessitated the need for locally derived reference equations from a sample of the general Nigerian population.

In addition, most of the available studies on pulmonary function tests in Nigeria were done several decades ago. However, as measurement techniques, equipment, and population characteristics evolve, there is a concurrent need to keep reference data up-to-date to reflect these changes [9].

This work was therefore aimed at providing up-to-date reference data for spirometric indices from a sample of the general adult Nigerian population.

## Methods

The survey was carried out in four (Asa, Ilorin East, Irepodun and Offa) out of the sixteen local government authority (LGA) in Kwara state from.

A cross sectional design aimed at obtaining representative sample of the population was used. Allowing for a 5% margin of error at 95% confidence interval and assuming that 60% of subjects will meet the 2005 ATS/ERS criteria [17] (as reported by the PLATINO survey [18]), the estimated sample size was about 640. However, a sample size of 720 subjects was recruited.

The surveyed local government authorities were selected by balloting. Participants were selected from the LGA headquarters and the nearest towns/villages to them. The selected sites were stratified according to the polling units and consecutive households were selected. All consenting individuals, in selected households, who fulfilled the selection criteria, were recruited until the sampling quota of each LGA was reached. The sampling quota was calculated based on the estimated population of the LGAs in the 2006 Census. Selected individuals who were unable to perform the spiromtery satisfactorily (see acceptability and repeatability criteria below) were replaced by newly selected individuals.

Healthy individuals who had never smoked cigarette were recruited if they were 18 years and above, able to perform spirometry satisfactorily, had no history of chronic illness such as sickle cell disease, without cardio respiratory symptoms or history of previously diagnosed cardio respiratory diseases and no history of abdominal or thoracic surgery/injury, eye surgery or heart attack in the last six months. They were without any evidence of cardio respiratory abnormality on physical examination.

Weight in kilograms (kg) was measured to the nearest 0.1 kg with subjects in minimal clothing without shoes on. Standing height in centimeter (cm) was measured to the nearest 0.1 cm without shoes, with both feet flat on the ground and apposed at the medial malleoli. The heels, buttocks and occiput were placed against the vertical bar, with the horizontally applied bar resting on the subject’s head.

Data collection was carried out by trained assistants under the supervision of the principal researcher (AEF). Interviewer administered questionnaire modeled on the American Thoracic society’s questionnaire was used. The initial part of the questionnaire contained questions on sociodemographic characteristics and evaluation to determine whether the subject was eligible for spirometry. The assistants were trained for two days and were required to perform at least 10 satisfactory spirometry before being selected for the survey. Spirometry was performed using Spiro lab III by MIR England. The spirometer has an auto sensor turbine for body temperature, pressure and water vapour saturated (BTPS) changes and so automatically correct spirometric reading for the body temperature and atmospheric pressure. The equipment was calibrated daily with a 3 l syringe. Measurements of spirometric indices were made in the upright (straight-backed) sitting position without a nose clip obstructing the nostrils. The subject to be tested was first made to be thoroughly familiar with the machine and the technique of the test. For FVC maneuver, the procedure was explained and demonstrated to the individual. The subject was told to sit straight with head slightly elevated, then inhaled maximally and rapidly, place the mouthpiece in the mouth and close the lips tightly around it, exhale maximally until no more air can be expelled while still maintaining an upright posture. The procedure was closely monitored to ensure it was acceptable. Thereafter the spirogram was examined for acceptability and repeatability criteria according to the ATS/ERS criteria [17]. A spirogram is acceptable if there is a satisfactory start of expiration (no hesitation), no air leak, no coughing during the procedure, no cessation of flow during the procedure and no evidence of extra breathing taken during the procedure. Measurement with an unacceptable start of test or an unusable curve was discarded before applying the repeatability criteria. The acceptable spirogram was also examined for repeatability criteria (the largest 2 values of FVC within 150mls of each other and the 2 largest values of FEV_1_ within 150mls of each other). If an individual achieved 3 acceptable readings that met the repeatability criteria, the session is concluded for the individual. If both criteria were not met, the testing was continued until the criteria were met or the individual was excluded from the survey after a total of 8 unacceptable tests have been performed. For VC procedure, the procedure was explained and demonstrated to the subject. The individual was instructed to completely fill and empty the lungs during the procedure which was performed in a relaxed manner (not forced) except near the end-inspiration and end-expiration. The subject was instructed to exhale completely, then, inhale completely and finally exhale completely through the mouthpiece. For the MVV procedure, it was explained and demonstrated to the subject. The individual was told to close the lips tightly around the mouthpiece and 3 resting tidal breaths were obtained, followed by breathing rapidly and deeply as possible for 12 s. Result was acceptable if it was performed with maximal effort without evidence of leakage, hesitation and tidal volume during the procedure was greater than the subjects resting tidal volume.

The largest VC, FVC, FEV_1,_ FEV_1_/FVC, FEV_3,_ FEV_6,_ MMEF_,_ PEFR_,_ FIVC, FIV_1_, PIF and MVV were recorded for each participant. All tests were performed between 9:00 am and 1:00 pm.

### Data analysis

Data analysis was done using the Statistical Package for Social Science software version 15.0. The data of height, age and lung function parameters was expressed as means ± SD for each sex separately. The distribution of the independent contributing variables was then examined for skewness and kurtosis to determine whether transformations will be necessary before performing regression analysis. Various regression models including quadratic, power functions, logtransformed and linear relationships were compared. For all lung indices examined, simple linear models provided more acceptable fits to the data. Therefore, linear models were chosen as the basic format for evaluating the relationships between the dependent variables and the independent variables.

Dependent variables (VC, FVC, FEV_1,_ FEV_1_/FVC, FEV_3,_ FEV_6,_ MMEF_,_ PEFR_,_ FIVC, FIV_1_, PIF and MVV) were regressed against height and age in different sex categories. They were first regressed individually against height and age. Stepwise multiple regression analyses were then used to determine which combination of variables would best fit the model. Predictor variables were retained in the regression model only if they significantly improved the explained variance of the dependent variable. The equations with the lowest residual standard deviations and highest coefficients of determination (R^2^) were considered acceptable, if each included variable contributed significantly to the model (*p* < 0.05). Lower limits of normal range were calculated as the lower fifth percentile of the distribution of the residuals from each equation.

In addition, spirometry of the population studied obtained by predicted equations of the present work and those of previous studies and the measured spirometry was compared using paired *t* test. A *p* value of < 0.05 was taken as significant.

## Results

Seven hundred and twenty participants made up of 358 males and 362 females who satisfactorily completed the spirometric measurements using the ATS/ERS [17] reproducibility and acceptability criteria were included in this analysis. Their age distribution is shown in Table 1. Majority of the males are in the 26–35 year age group whereas the majority of the females are in the 56–65 year age group.

**Table 1:** Age distribution (in years) of the participants

Age group	Males (358)	Females (362)
	Frequency (%)	Frequency (%)
18–25	81(22.6)	87(24.0)
26–35	95(26.5)	44(12.2)
36–45	64(17.9)	71(19.6)
46–55	50(14.0)	43(11.9)
56–65	68(19.0)	117(32.3)

Anthropometric characteristics and spirometric parameters of the male and female participants are summarized in Table 2. The mean, median, skewness and kurtosis of the parameters indicated that regression was possible without need for data transformation. The males are younger, taller and have higher values of spirometric indices (except for FEV_1_/FVC) than the females.

**Table 2:** Anthropometric and spirometric parameters in male and female participants

Parameters	Range	Mean	SD	Median	Skewness	Kurtosis
Males(*N* = 358)						
Age (years)	18–65	38.3	14.9	37.0	0.529	−1.038
Height (cm)	155.0–185.0	170.7	8.0	170.0	−0.140	−1.120
VC (Liters)	2.71–5.80	4.10	0.81	4.00	0.162	−0.733
FVC (Liters)	2.42–4.99	3.81	0.73	3.98	−0.297	−1.091
FEV_1_ (Liters)	2.10–4.39	3.37	0.67	3.46	−0.213	−1.092
FEV_1_/FVC (%)	77.6–95.2	88.4	4.7	89.4	−0.526	−0.621
FEV_3_ (Liters)	2.41–4.59	3.69	0.70	3.78	−0.296	−1.273
FEV_6_ (Liters)	2.42–4.98	3.81	0.73	3.98	−0.297	−1.091
MMEF (Liters/sec)	2.65–7.25	4.70	1.23	4.42	0.423	−0.676
PEFR (Liters/sec)	5.62–12.50	9.29	1.71	9.16	−0.191	−0.575
MVV (Liters/min)	73.5–212.4	137.7	31.9	139.9	−0.750	−0.281
FIVC (Liters)	2.11–4.86	3.56	0.68	3.48	−0.161	−0.645
FIV_1_ (Liters)	1.92–4.31	3.29	0.63	3.28	−0.206	−0.625
PIF (Liters/sec)	5.59–10.72	7.86	1.20	7.99	−0.492	−0.322
Females (*N* = 362)						
Age (years)	18–63	42.5	15.4	42.0	- 0.148	−1.496
Height (cm)	143.0–172.0	156.8	6.1	158.0	−0.023	0.069
VC (Liters)	1.64–5.08	2.65	0.72	2.64	1.362	3.218
FVC (Liters)	1.73–3.25	2.52	0.47	2.67	−0.327	−1.275
FEV_1_ (Liters)	1.46–3.00	2.24	0.44	2.25	−0.179	−1.044
FEV ratio (%)	77.6–98.9	89.5	5.74	89.3	−0.152	−0.410
FEV_3_ (Liters)	1.64–3.25	2.45	0.46	2.56	−0.295	−1.171
FEV_6_ (Liters)	1.73–3.25	2.52	0.47	2.66	−0.328	−1.278
MMEF (Liters/sec)	1.96–6.30	3.66	1.10	3.56	0.579	−0.278
PEFR (Liters/sec)	3.56–8.33	5.91	1.24	5.90	0.183	−0.543
MVV (Liters/min)	57.6–120.0	89.6	15.2	93.1	−0.126	−0.878
FIVC (Liters)	1.55–3.01	2.39	0.49	2.62	−0.462	−1.303
FIV_1_ (Liters)	1.32–2.68	2.12	0.46	2.38	−0.454	−1.368
PIF (Liters/sec)	3.05–7.15	5.19	0.94	5.30	−0.315	−0.124

*SD* standard deviation, *VC* vital capacity, *FVC* forced vital capacity, *FEV*_*1*_ forced expiratory volume in one second, *FEV ratio* FEV1/FVC%, *FEV*_*3*_ forced expiratory volume in three seconds, *FEV*_*6*_ forced expiratory volume in six seconds, *PEFR* peak expiratory flow rate, *MMEF* maximal mid expiratory flow, *MVV* maximal voluntary ventilation, *FIVC* forced inspiratory vital capacity, *FIV*_*1*_ forced inspiratory volume in one second, *PIF* peak inspiratory flow

**Fig. 1:**
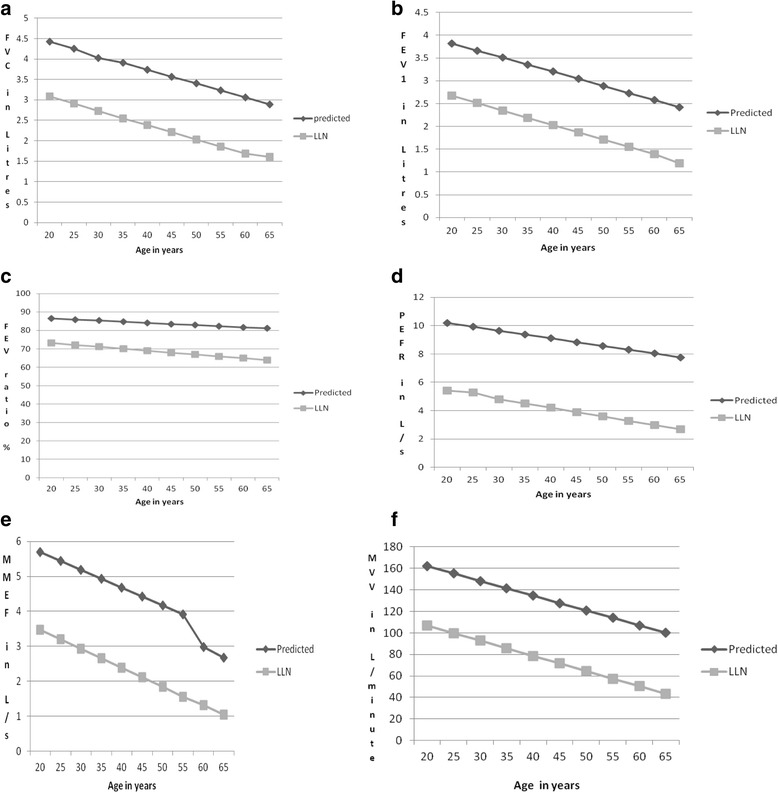
**a**-**f** Predicted mean and lower limit of normal (LLN) for FVC, FEV_1_, FEV ratio, PEFR, MMEF and MVV in males (height 170 cm) by age. FVC = forced vital capacity (Liters), FEV_1_ = forced expiratory volume in one second (Liters), FEV ratio = FEV1/FVC%, PEFR = peak expiratory flow rate(Liters/s), MMEF = maximal mid expiratory flow (Liters/s), MVV = maximal voluntary ventilation (Liters/min)

**Fig. 2:**
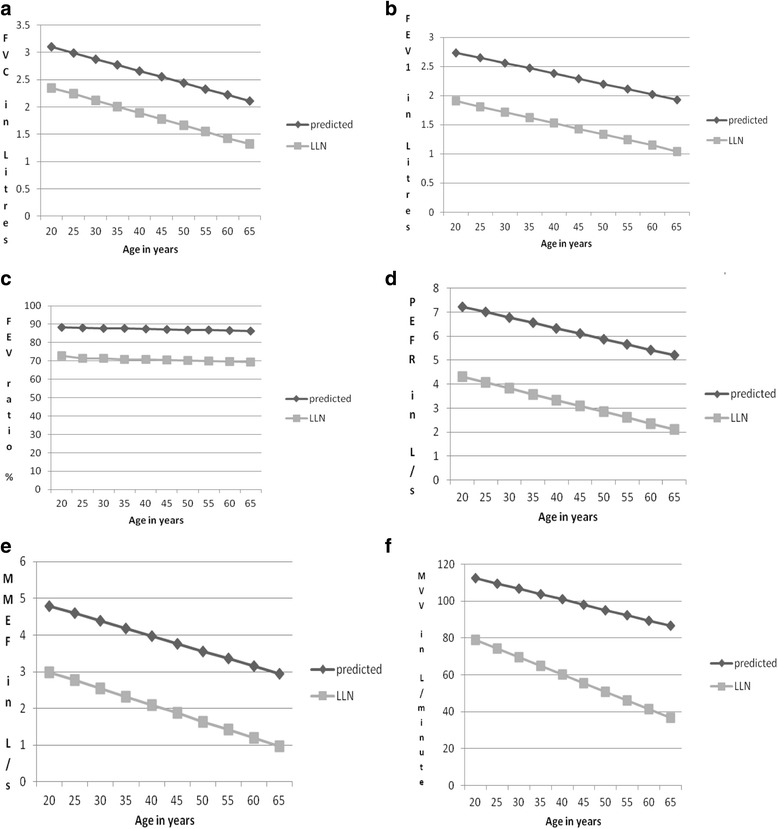
**a**-**f** Predicted mean and lower limit of normal (LLN) for FVC, FEV_1_, FEV ratio, PEFR, MMEF and MVV in females (height 165 cm) by age. FVC = forced vital capacity (Liters), FEV_1_ = forced expiratory volume in one second (Liters), FEV ratio = FEV1/FVC%, PEFR = peak expiratory flow rate(Liters/s), MMEF = maximal mid expiratory flow (Liters/s), MVV = maximal voluntary ventilation (Liters/min)

The prediction equations derived for the various spirometric parameters after multiple regression analysis are described in Table 3. All the parameters correlated negatively with age and positively with height. Figures 1 and 2 demonstrate the linear fall of the spirometric parameters with age.

**Table 3:** Derived equations for various spirometric parameters of male and female subjects

Males	
VC (R^2^ = 0.745, RSD = 0.41225)	
Predicted	˗1.163 + (-0.035)A + 0.039H
LLN	˗2.148 + (-0.037)A + 0.034H
FVC (R^2^ = 0.837, RSD = 0.29524)	
Predicted	˗ 0.848 + (-0.034)A + 0.035H
LLN	˗1.487 + (-0.035)A + 0.031H
FEV_1_ (R^2^ = 0.821, RSD = 0.28328)	
Predicted	˗0.834 + (-0.031)A + 0.031H
LLN	˗1.448 + (-0.032)A + 0.028H
FEV ratio (R^2^ = 0.08, RSD = 4.6897)	
Predicted	82.448 + (-0.120)A + 0.038H
LLN	72.288 + (-0.206)A + (0.029)H
FEV_3_ (R^2^ = 0.845, RSD = 0.27565)	
Predicted	˗0.613 + (-0.033)A + 0.033H
LLN	˗1.210 + (-0.034)A + 0.029H
FEV_6_ Same as for FVC	
MMEF (R^2^ = 0.450, RSD = 0.91509)	
Predicted 3.830 + (-0.051)A + 0.017H	
LLN	1.997 + (-0.054)A + 0.015H
PEFR (R^2^ = 0.573, RSD = 1.11787)	
Predicted	˗4.199 + (-0.054)A + 0.091H
LLN	˗6.621 + (-0.061)A + 0.078H
MVV (R^2^ = 0.594, RSD = 20.3577)	
Predicted	34.221 + (-1.378)A + 0.915H
LLN	20.883 + (-1.408)A + 0.671H
FIVC (R^2^ = 0.707, RSD = 0.37060)	
Predicted	˗1.407 + (-0.027)A + 0.035H
LLN	˗2.210 + (-0.029)A + 0.031H
FIV_1_ (R^2^ = 0.661, RSD = 0.36884)	
Predicted	˗1.572 + (-0.023)A + 0.034H
LLN	˗2.371 + (-0.025)A + 0.029H
PIF (R^2^ = 0.458, RSD = 0.88486)	
Predicted	˗2.366 + (-0.029)A + 0.066H
LLN	˗4.283 + (-0.035)A + 0.056H
Females	
VC (R^2^ = 0.688, RSD = 0.40299)	
Predicted	˗2.980 + (-0.029)A + 0.044H
LLN	˗3.975 + (-0.032)A + 0.038H
FVC (R^2^ = 0.876, RSD = 0.16623)	
Predicted	˗1.578 + (-0.022)A + 0.032H
LLN	˗1.988 + (-0.023)A + 0.030H
FEV_1_ (R^2^ = 0.761, RSD = 0.21655)	
Predicted	˗1.861 + (-0.018)A + 0.031H
LLN	˗2.195 + (-0.019)A + 0.028H
FEV ratio (R^2^ = 0.034, RSD = 5.6592)	
Predicted	60.815 + (-0.044A) + 0.177H
LLN	48.554 + (-0.054A) + 0.152H
FEV_3_ (R^2^ = 0.860, RSD = 0.17413)	
Predicted	˗2.374 + (-0.020)A + 0.036H
LLN	˗2.804 + (-0.021)A + 0.033H
FEV_6_ (R^2^ = 0.876, RSD = 0.16555)	
Predicted	˗1.521 + (-0.022)A + 0.032H
LLN	˗1.930 + (-0.023)A + 0.029H
MMEF (R^2^ = 0.552, RSD = 0.73682)	
Predicted	˗3.830 + (-0.041)A + 0.059H
LLN	˗5.548 + (-0.045)A + 0.059H
PEFR (R^2^ = 0.611, RSD = 0.77743)	
Predicted	˗4.986 + (-0.045)A + 0.082H
LLN	˗5.903 + (-0.049)A + 0.070H
MVV (R^2^ = 0.550, RSD = 10.2106)	
Predicted	3.752 + (-0.578)A + 0.752H
LLN	5.102 + (-0.939)A + 0.579H
FIVC (R^2^ = 0.676, RSD = 0.27872)	
Predicted	˗1.201 + (-0.020)A + 0.028H
LLN	˗1.888 + (-0.022)A + 0.024H
FIV_1_ (R^2^ = 0.575, RSD = 0.30378)	
Predicted	˗0.762 + (-0.018)A + 0.023H
LLN	˗1.511 + (-0.020)A + 0.019H
PIF (R^2^ = 0.564, RSD = 0.62244)	
Predicted	˗3.928 + (-0.030)A + 0.066H
LLN	˗5.464 + (-0.034)A + 0.057H

*R* ^*2*^ coefficient of determination, *RSD* residual standard deviation, *LLN* lower limit of normal, *A* age in years, *H* height in centimetres, *VC* vital capacity (Liters), *FVC* forced vital capacity (Liters), *FEV*_*1*_ forced expiratory volume in one second (Liters), *FEV ratio* FEV1/FVC%, *FEV*_*3*_ forced expiratory volume in three seconds (Liters), *FEV*_*6*_ forced expiratory volume in six seconds (Liters), *PEFR* peak expiratory flow rate(Liters/sec), *MMEF* maximal mid expiratory flow (Liters/sec), *MVV* maximal voluntary ventilation (Liters/min), *FIVC* forced inspiratory vital capacity (Liters), *FIV*_*1*_ forced inspiratory volume in one second (Liters), *PIF* peak inspiratory flow (Liters/sec)

A comparison of spirometric values derived by prediction equations of the present study with mean measured values and those of various other studies in Nigeria and other parts of the world are presented in Table 4. It shows that our values are higher than values obtained from previous studies from Nigeria but the difference is, however, not statistically significant. Likewise, there are no statistically significant differences between values from our equations and those from study done in Ethiopia. Our FVC and FEV_1_ values are, however, significantly lower than values from Iran. Values from Poland are also significantly higher than our values. We also compared values from our equations with the values derived for Afro Americans in the landmark global lung function initiative study. Our maximal mid expiratory flow values were significantly higher in both males and females and in addition our female FVC values were lower than the values for the Afro Americans. Our FEV_1_/FVC was higher than the values for the Afro Americans although the difference was not statistically significant.

**Table 4:** Comparison between spirometric values obtained by predicted equations of the present study with mean measured values and those of previous studies

Parameters	Age range (years)	Subjects (No)	FVC (Litres)	FEV1 (Litres)	Ratio (%)	MMEF (Litres/second)	PEFR (Litres/second)
Male (age = 38.5 years and height = 170.7)							
Predicted present study	18–65	358	3.82	3.27	84.3	4.78	9.27
Mean present study	18–65	358	3.81	3.37	88.4	4.70	9.29
SD			*p* = 0.97	*p* = 0.69	*p* = 0.53	*p* = 0.82	*p* = 0.98
Elebute et al [14]	17–54	142	--	--	--	--	8.08
SD			--	--	--	--	*p* = 0.18
Njoku et al [15]	15–60	668	-	-	-	-	8.84
SD			-	- -		-	*p* = 0.47
Erhabor et al [16]	19–60	241	3.59	3.00	84.6	--	8.71
SD			*p* = 0.46	*p* = 0.31	*p* = 0.97	--	*p* = 0.30
Mengesha et al [23]	18–47	143	4.48	3.62	--	--	8.48
SD			*p* = 0.10	*p* = 0.29	--	--	*p* = 0.63
Golshan et al. [24]	21–82	1302	4.59	3.97	86.5	5.12	10.20
SD			*p* = 0.004*	*p* = 0.002*	*p* = 0.67	*p* = 0.23	*p* = 0.12
Ostrowski et al. [26]	40–80	49	5.50	4.45	--	--	--
SD			*p* = 0.007*	*p* = 0.02*	--	--	--
GLI Afro American [11]	3–95	1520	4.05	3.33	82.0	3.40	
SD			*p* = 0.33	*p* = 0.76	*p* = 0.63	*p* < 0.01*	
Female (age = 42.5 and height = 156.8)							
Predicted present study	18–65	362	2.51	2.23	86.7	3.68	5.96
Mean present study	18–65	362	2.52	2.24	89.5	3.66	5.91
SD			*p* = 0.96	*p* = 0.95	*p* = 0.67	*p* = 0.94	*p* = 0.91
Elebute et al [14]	17–44	88	--	--	--	--	5.60
SD			--	--	--	--	*p* = 0.61
Njoku et al [15]	15–60	341	-	-	-	-	5.99
SD			-	-	-	-	*p* = 0.95
Mengesha et al [23]	18–47	117	2.94	2.30	--	--	--
SD			*p* = 0.12	*p* = 0.77	--	--	--
Golshan et al [24]	21–80	1110	3.14	2.74	87.6	3.64	6.77
SD			*p* < 0.001*	*p* < 0.01*	*p* = 0.87	*p* = 0.86	*p* = 0.04*
Ostrowski et al [26]	40–80	87	3.45	2.93	--	--	--
SD			*p* = 0.004*	*p* = 0.01*	--	--	--
GLI Afro American [11]	3–95	2025	2.87	2.36	83.0	2.58	
SD			*p* = 0.03*	*p* = 0.33	*p* = 0.44	*p* < 0.01*	

*FVC* forced vital capacity, *FEV*_*1*_ forced expiratory volume in one second, *ratio* FEV1/FVC%, *PEFR* peak expiratory flow rate, *MMEF* maximal mid expiratory flow, *GLI* global lung finction initiative study, *SD* statistical significance, * = statistically significant difference

## Discussion

Measurements of ventilatory function are not only important as a diagnostic tool; they are also useful in following the natural history of disease over a period of time, assessing the preoperative risk and in quantifying the effects of treatment. Spirometry is very important in the field of respiratory care because it is able to identify obstructive pathology about 5–15years prior to the other techniques [19].

Ethnicity has been recognized as one of the major factors responsible for variability of lung function [2–4, 20, 21]. It is therefore important to establish reference values relevant to the ethnic characteristics of local population. Changes in population dynamics also necessitate the need for periodic review of such established reference values [22].

We used linear regression models to obtain equations for reference values of spirometric indices for adult Nigerians from a sample of the general population aged 18–65 years (males) and 18–63 years (females). As found in many other studies [3, 6, 12, 15, 23], the current study found that the most important predictive variables were height and age and linear equations performed satisfactorily.

Our study shows that the values of the spirometic indices increase with increasing stature but decrease with increasing age in both sexes as reported in previous studies [15, 23]. The sex difference in all the indices is also apparent as all the indices, except FEV_1_/FVC, are higher in men than in women. This trend had been reported earlier. The reason for this is not an objective of this study but previous studies have suggested differences in muscular strength, size and shape of the thoracic cage and elasticity of the lung as possible reasons for the observed sex difference in values of spirometric indices [23–25].

When comparison was made using a standard age and height as shown in Table 4, our results show that our values are higher than values obtained from previous studies in Nigeria (except FEV_1_/FVC) but the differences were not statistically significant [16]. This suggests that although the values are increasing, the increase is yet to be significantly different from values obtained using the past equations. The implication of this is that there is need for periodic study to derive new equations so as to recognize when there is significant difference.

We also compared values of PEFR derived from our linear equations with the curvilinear equations from Njoku et al. [15] and found out that although our values are higher, the difference was not statistically significant even at both ends of the age distribution. This shows that our linear equations performed satisfactorily for the age range of the population studied. Similarly, there was no significant difference between values from our equations and those obtained from study among Ethiopians [23]. This is in agreement with the previous studies on spirometric parameters in Nigeria and other populations of African descent [14–16]. Compared to report from Iran, our FVC and FEV_1_values (in males and females) as well as PEFR (in females) are significantly lower [24]. Our values are also lower than values from Poland [26]. The differences obtained in these indices among the ethnic groups may be attributable to complex interrelationship of factors such as genetic and physical make up of the people in the ethnic groups, altitude, environmental differences, physical activities, and tobacco smoking [2–4, 20–22, 27]. This is why it is important to always interpret ventilatory function tests in any individual by comparing it with reference values obtained from a well-defined population of healthy subjects of the same ethnic origin. Similar to an earlier report from Tunisia [28], we observed disparities between values obtained from our equations and those for Afro Americans using the GLI equations [11]. These disparities may be due to many reasons including substantial differences in anthropometric, socioeconomic and environmental factors. It suggests that the use of the multi-ethnic GLI equations for Nigerians may lead to misinterpretation of spirometry data especially among the females and this may result in inappropriate diagnosis and/or management. This is in agreement with a Swedish study, by Backman et al. [29], which reported that the use of GLI reference values may produce biased prevalence estimates of lung diseases in Sweden, especially among women.

One of the limitations of our study is that the participants are from kwara state only and so it may not represent the true picture of the whole country. However, the fact that the participants were selected from the general population is an important improvement over previous studies which selected participants from non representative groups such as students, health workers, males’ only etc. [12–16]. Another drawback of our study is that it included participants with age range 18-65years unlike the current trend of trying to include people of all ages in spirometric reference equations [11]. In spite of the recent endorsement and calls for the adoption of the GLI reference values in clinical practice worldwide [30, 31], our study is still very relevant in Nigeria context because the GLI study did not include data from Nigeria and in addition spirometry is being done in few centers in Nigeria almost entirely in adults only. Our spirometric data were collected without the use of nose clips in the subjects because majority of them objected to its use. This is likely to have affected our results especially the inspiratory parameters.

Our prediction equations for spirometry indices fill a serious lacuna in an important aspect of pulmonary care in Nigeria for many important reasons. First of all, it is the first study to describe the derivation of spiromteric reference values for Nigerians from a sample of the general population unlike previous studies which selected participants from non representative groups. Another point in favour of our study is its size and representativeness, including a substantial proportion of people in older age groups, who were underrepresented in previous studies. The sample size is fairly well distributed over the age group in this study because we went to the communities to select our participants and this may have positive influence on the accuracy of our equations. However, caution is required in interpreting the prediction equations for the elderly because of possible bias as a result of error in age. Many elderly individuals in Nigeria do not have proper records of their date of birth and usually estimate it by events which occurred around the time of their birth.

Also our study has provided prediction equations for spirometric indices such as VC, FEV_1_/FVC, FEV_3_, FEV_6_, MMEF, FICV, FIV_1_ and PIF which were not included in previous studies from Nigeria. Some of the indices are now being considered as important variables to use in some special situations. For example, in obstructive lung disease, the FVC may be less than the slow VC because of earlier closure during the forced manoeuvre. This may lead to an overestimation of the FEV_1_/FVC. Thus the FEV_1_/VC may be a more sensitive index of airflow obstruction [19]. Furthermore, FEV_1_/FEV_6_ and FEV_6_ have been suggested as alternative to FEV_1_/FVC and FVC in the spirometric detection of airway obstruction and restriction [32].

Another strength of our study is that it is the first study, in Nigerians, to provide prediction equations for the lower limit of normal (LLN) for spirometric parameters. The LLN derived directly from the population provides more accurate estimates than assuming a constant value of less than 80% of the predicted as being low. For example, Hardie et al. [33] have shown that the use of a fixed FEV_1_/FVC cut-off point of 70% for defining chronic obstructive pulmonary disease (COPD), as recommended by the Global Initiative for COPD, may lead to significant degree of overdiagnosis of both the presence and severity of COPD in the elderly population. In agreement with that study, our present prediction equations indicate that a lower FEV_1_/FVC should be used in the elderly. Based on our prediction equations, a 65 year old male with a height of 170 cm has a LLN for FEV_1_/FVC of 63.9% and this is expected to be even lower for shorter individuals. The dependency of LLN of spirometric parameters on age does not only affect the FEV_1_/FVC. As shown in Figs. 1 and 2, the LLN of all the indices is dependent on age. Although both ATS and the ERS accept the use of the 5^th^ percentile to define the LLN (which unlike the percentage of the predicted, is free from bias due to age, height, sex or ethnic group), some authors have observed that a substantial percentage of subjects (±10.4%) have values lower than the 5^th^ percentile [1, 9, 34].

Interaction with colleagues in centres with pulmonary laboratories in Nigeria reveal that most of them rely on the default settings for predicted values for different ranges offered by the manufacturer and it seems as if many of them are still unaware of the problems associated with such practices, especially the use of fixed percentage as LLN. We advocate for the use of our new prediction equations in order to help in surmounting this great barrier to proper care of our patients with respiratory disorders.

## Conclusion

We present the findings from our study, the first of such to select representative sample from the general adult population, the first to provide equations for some parameters which were not included in previous equations and also the first to provide equations for the LLN in Nigerians. Our new prediction equations for spirometric indices are a significant improvement over the previous Nigerian equations. The use of our prediction equations is expected to ensure that the prevalence estimates of adult with spirometric parameters below the LLN are possible using the LLN equations derived from the general Nigerian population rather than assuming a value below 80% as is currently done. Our study also has unequivocal clinical implications because everyday decisions are made with regard to diagnosis, treatment and prevention of respiratory conditions on the basis of spirometric results in relation to reference values.

## Abbreviations

ATS/ERS: American thoracic society/European respiratory society
BTPS: Body temperature, pressure and water vapour saturated
FEV ratio: FEV1/FVC%
FEV_1_: Forced expiratory volume in one second
FEV_3_: Forced expiratory volume in three seconds
FEV_6_: Forced expiratory volume in six seconds
FIV_1_: Forced inspiratory volume in one second
FIVC: Forced inspiratory vital capacity
FVC: Forced vital capacity
MMEF: Maximal mid expiratory flow
MVV: Maximal voluntary ventilation
PEFR: Peak expiratory flow rate
PIF: Peak inspiratory flow
VC: Vital capacity
